# Bile salt hydrolase: a key player in gut microbiota and its implications for metabolic dysfunction-associated steatotic liver disease

**DOI:** 10.20517/mrr.2025.12

**Published:** 2025-07-30

**Authors:** Weixing Zhao, Huiying Wang, Minghua Zheng, Yan Ni

**Affiliations:** ^1^Children’s Hospital, Zhejiang University School of Medicine, National Clinical Research Center for Child Health, Hangzhou 310052, Zhejiang, China.; ^2^Department of Epidemiology and Biostatistics, School of Public Health, Zhejiang University, Hangzhou 310058, Zhejiang, China.; ^3^MAFLD Research Center, Department of Hepatology, the First Affiliated Hospital of Wenzhou Medical University, Wenzhou 325000, Zhejiang, China.; ^#^Authors contributed equally.

**Keywords:** Bile salt hydrolase, gut microbiota, bile acids, metabolic dysfunction-associated steatotic liver disease

## Abstract

The rising prevalence of metabolic dysfunction-associated steatotic liver disease (MASLD) poses a significant global public health challenge. Bile acids (BAs), synthesized in the liver and further metabolized in the gut, are essential in maintaining host metabolic homeostasis. Bile salt hydrolase (BSH), an enzyme produced by the gut microbiota, catalyzes the hydrolysis of conjugated BAs, thus regulating the balance between primary and secondary BAs. Growing evidence suggests that BSH activity is intricately linked to the pathogenesis of MASLD. This review comprehensively examines the structural and functional properties of BSH enzymes, their distribution among gut microbial communities, and current methodologies for assessing BSH expression and activity. Furthermore, it highlights the alterations in BSH observed in MASLD and explores the potential mechanistic pathways involved, offering a foundation for the development of novel diagnostic and therapeutic strategies.

## INTRODUCTION

Metabolic dysfunction-associated steatotic liver disease (MASLD) is the most common chronic liver disease globally, characterized by hepatic fat accumulation that can progress from simple steatosis [metabolic dysfunction-associated steatosis (MASL)] to steatohepatitis [metabolic dysfunction-associated steatohepatitis (MASH)], fibrosis, and even cirrhosis and hepatocellular carcinoma^[1]^. MASLD is linked not only to liver-related complications but also to extrahepatic manifestations such as cardiovascular disease, chronic kidney disease, and certain cancers^[2]^. With a global prevalence of approximately 38.77%, MASLD has become a major global public health concern, imposing a heavy burden on individuals and society^[3]^. Therefore, the development of effective strategies for the prevention, screening, and treatment of MASLD is of great importance^[4]^.

Bile acid (BA) metabolism and its interaction with the gut microbiota play important roles in modulating host immunity and influencing the pathogenesis of MASLD^[5,6]^. Primary bile acids (PBAs) are synthesized from cholesterol in the liver via both classical and alternative pathways. These PBAs are conjugated with taurine or glycine to form conjugated bile acids (conBAs), which are stored in the gallbladder and released into the intestine upon food intake to facilitate lipid digestion. In the intestine, the gut microbiota converts PBAs into secondary bile acids (secBAs), primarily deoxycholic acid (DCA) and lithocholic acid (LCA). Both PBAs and secBAs can activate receptors such as the farnesol X receptor (FXR) and G protein-coupled bile acid receptor 1 (TGR5), which regulate glucose and lipid metabolism as well as immune responses^[7]^.

During the microbial transformation of BAs, certain gut bacteria that express bile salt hydrolase (BSH) enzymes can deconjugate conBAs into unconjugated bile acids (unconBAs), initiating further modifications such as dehydroxylation and epimerization^[5]^. As such, BSH is often regarded as the “gatekeeper” of BA modifications in the gut^[8]^, with significant influence on host metabolic processes including lipid digestion and cholesterol metabolism. Recent studies have highlighted close correlations between BSH activity and metabolic diseases, suggesting that BSH could serve as a therapeutic target in MASLD^[9-11]^. This review provides an overview of the structural features and functions of BSH, its distribution among gut bacteria, and current evaluation methods. It also examines changes in microbial BSH activity during the progression of MASLD, offering insights into the complex relationship between BSH, BA metabolic balance, and MASLD pathogenesis, with the goal of identifying novel therapeutic strategies for this widespread disease.

## BILE SALT HYDROLASE

### Molecular structure of BSH

BSH, classified as EC 3.5.1.24 in the Kyoto Encyclopedia of Genes and Genomes (KEGG) database, is an enzyme that hydrolyzes the amide bond in conjugated bile acids, converting them into free bile acids and amino acids. It belongs to the N-terminal nucleophile (Ntn) hydrolase family and typically consists of 314-338 amino acids, with a monomeric molecular weight of approximately 34-42 kDa. The enzyme’s active sites include Cys2, Arg18, Asp21, Asn175, and Arg228, with the cysteine residue being highly conserved^[12]^. BSH generally exists as a homotetramer, although other homooligomeric forms such as hexamers or octamers have also been observed. Notably, structural variations can occur even within the same organism, suggesting that different BSH isoforms may exhibit distinct substrate specificities^[13]^. For instance, recent research has shown that BSH enzymes from *Lactobacillus* species possess two different substrate selectivity loops: those with G-V/T-G motifs demonstrate a higher affinity for taurine-conjugated BAs, while those with S-R-G/S motifs are more selective for glycine-conjugated BAs^[8]^. This structural diversity highlights the important role of BSH in regulating BA modification based on substrate specificity.

### Metabolic function of BSH

BSH plays a pivotal role in BA metabolism by deconjugating bile salts, thereby altering their physicochemical properties and influencing their absorption and reabsorption in the intestine. Deconjugation is a key step in the enterohepatic circulation of BAs, as it affects the composition of the BA pool and impacts processes such as lipid emulsification, cholesterol metabolism, and gut microbial ecology. BSH is traditionally understood to function by hydrolyzing amide bonds in conBAs, releasing free BAs and their respective amino acid residues^[5]^. However, recent studies have uncovered that BSH can also catalyze the conjugation of unconBAs with amines to form bacterial bile acid amidates (BBAAs). This discovery challenges the conventional understanding of BSH functions and provides new insights into its broader role in BA metabolism^[14]^.

### Distribution of BSH in the gut microbiota

BSH is widely distributed among gut bacteria species, with activity levels varying depending on bacterial strain and environmental conditions. BSH genes have been identified in *Lactobacillus*, *Bifidobacterium*, *Clostridium*, *Enterococcus, Bacteroides, Listeria*, *Brucella*, and *Xanthomonas*, which are typically associated with high BSH activity^[15]^. Advances in next-generation sequencing and bioinformatics have significantly enhanced our understanding of the BSH gene in the human gut microbiome. These studies indicate that BSH genes are predominantly expressed in members of the phyla *Firmicutes*, *Bacteroidetes*, *Actinobacteria*, *Proteobacteria*, and *Euryarchaeota*, with *Lactobacillus* species showing the highest BSH activity in the human gut^[9,16]^. Despite these findings, the structural and functional diversity of BSH enzymes across different microbial taxa remains insufficiently understood.

### Evaluation methods for changes in gut microbial BSH

The expression and activity of microbial BSH are typically evaluated by measuring changes in the composition and concentration of the BA pool, BSH gene expression levels, or *in vitro* enzymatic activity. Four common tools are used for these measurements: mass spectrometry, ninhydrin colorimetry, microbial sequencing, and activity probes [Table 1]. Mass spectrometry offers high sensitivity and specificity, enabling the precise quantification of BSH activity by measuring the concentrations of conBAs and unconBAs to determine the hydrolysis rate^[17,18]^. Ninhydrin colorimetry is a traditional method that detects amino acids released by BSH activity through a colorimetric reaction^[19]^. Although less specific and accurate than modern techniques, it remains a simple and cost-effective approach for evaluating enzyme activity *in vitro*. Microbial sequencing can infer changes in BSH expression by analyzing homologous gene sequences, copy numbers, or the relative abundance of BSH-expressing bacterial taxa^[9-11,16,20-22]^. Moreover, recent developments in activity-based probes have significantly improved the precision of BSH activity measurement^[23,24]^. Probes such as Ch-AOMK bind specifically to BSH active sites and enhance quantitative analysis via mass spectrometry. These probes hold great potential for non-invasive diagnostics and therapeutic monitoring in conditions such as inflammatory bowel disease (IBD)^[24]^.

**Table 1:** Evaluation methods for intestinal BSH activity

**Method**	**Target compounds/microbes**	**Sample**	**BSH activity indicators**	**Pros and cons**	**References**
Mass spectrometry	Bile acid profiles (e.g., GCDCA, CDCA)	Cecal contents, serum, feces	Ratio of CDCA to TCDCA	Pros: high accuracy, high throughput Cons: expensive	[17,18]
Ninhydrin colorimetry	Free amino acids released from conjugated BAs	Feces	Rate of CA generation	Pros: cost-effective Cons: low specificity	[19]
Microbial sequencing					
16s rRNA	*Bacteroides, Clostridium, Lactobacillus*	Feces	Relative abundance of representative BSH-producing bacteria	Pros: established method Cons: indirect reflection of BSH gene expression	[20]
	*Bacteroides, Lactobacillus, Bifidobacterium*				[21]
	*Bifidobacterium, Lactobacillus*				[22]
16s rRNA	Various species	Feces	Relative abundance of BSH genes	Pros: easy to perform Cons: limited by database coverage and update frequency	[9,10]
Metagenomics	Various species	Feces	Relative abundance of BSH homologous sequences	Pros: high accuracy, comprehensive analysis Cons: poor data quality and parameter consistency	[9,16]
Activity probe	Ch-AOMK, BAL	Bacteria, feces	Fluorescence intensity	Pros: high sensitivity Cons: expensive	[23,24]

BSH: Bile salt hydrolase; CDCA: chenodeoxycholic acid; BAs: bile acids; GCDCA: glycochenodeoxycholic acid; TCDCA: taurochenodeoxycholic acid; CA: cholic acid; BAL: BSH-activatable luciferin.

## CHANGES IN MICROBIAL BSH IN MASLD

Microbial BSH plays a pivotal role in the pathophysiology of MASLD. MASLD is characterized by excessive fat accumulation in the liver, and recent evidence suggests that alterations in gut microbial BSH may influence disease progression through modulation of BA metabolism. This section summarizes the changes in microbial BSH across the disease spectrum, from MASLD to its more severe forms including MASH and liver fibrosis [Table 2].

**Table 2:** Changes in BSH expression or activity across different stages of MASLD

**BSH change^a^**	**Subjects**	**Ethnicity/animal model**		**Intervention**	**Group**	**BSH activity evaluation method**	**Microbiome sequencing method**	**References**
Metabolic dysfunction-associated steatotic liver disease (MASLD)								
↓	Adults	American		/	mild MASLD (72) *vs.* Control (308)	BSH gene abundance	Metagenomics	[9,59,60]
↓	Children	Asian		/	MASLD (32) *vs.* HC (36)	Microbial relative abundance, KEGG analysis	Metagenomics	[25]
↑	Adults	American		/	MASLD (57) *vs.* HC (18)	Microbial relative abundance	16s rRNA	[11]
↓	Mice	C57BL/6J		/	HFD (6) *vs.* Chow (9)	Microbial relative abundance	16s rRNA	[22]
↓	Mice	C57BL/6J		Drug	HFD + IsA (6) *vs.* Chow *vs.* HFD (6)	*In vitro* assay	16s rRNA	[28]
↓	Mice	C57BL/6J		Drug	HFD (8) *vs.* CN (8)	Microbial relative abundance	16s rRNA	[29]
↓	Mice	C57BL/6J		Drug	ZKY (6) *vs.* HFD (6) *vs.* Con (6）	BSH gene copy number	16s rRNA	[26]
↑	Rats	Wistar		BSH inhibitor	AAA-10 (8) *vs.* Vehicle (8)	*In vitro* assay	/	[31]
↑	Mice	C57BL/6J		BSH inhibitor	CAPE (5) *vs.* Vehicle (5)	*In vitro* assay	16s rRNA	[17]
↑	Mice	C57BL/6J		Drug	PCPE (8) *vs.* HFD (8)	*In vitro* assay	16s rRNA	[30]
Metabolic-Associated Steatohepatitis (MASH)								
↓	Mice	C57BL/6J		Probiotics	ALDH2-/--MCD (5) *vs.* WT-MCD (5)	*In vitro* assay	16s rRNA	[32]
↓	Mice	C57BL/6J		Drug	OCA (8) *vs.* Vehicle (8)	*In vitro* assay	16s rRNA	[34]
↑	Mice	C57BL/6J		/	HFHC (8) *vs.* HF (8) *vs.* Con (8)	Microbial relative abundance	16s rRNA	[33]
Advanced liver diseases (Fibrosis and cirrhosis)								
↓	Adults	Asian	/		Hepatic fibrosis (17) *vs.* HC (10)	Microbial relative abundance, bile acid profile analysis	16s rRNA	[18]
↓	Adults	American	/		Advanced MASLD (Fibrosis,14) *vs.* Control (308)	BSH gene abundance	Metagenomics	[9,59,60]
↓	Adults	Asian	/		Liver cirrhosis (114) *vs.* Control (123)	BSH gene abundance	Metagenomics	[9,38]
↓	Mice	C57BL/6J	Probiotics		TAA (6) *vs.* Control (6)	Bile acids profile analysis	16s rRNA	[18]
↓	Mice	C57BL/6J	/		Tlr4-/- (8) *vs.* WT (10)	Microbial relative abundance	16s rRNA	[36]
↓	Mice	C57BL/6J	Drug		DDC + KH (5) *vs.* DDC+H2O (5)	Microbial relative abundance	16s rRNA	[35]
↓	Mice	C57BL/6J	Drug		DDC (5) *vs.* Control (5)	*In vitro* assay	16s rRNA	[19]
↓	Mice	C57BL/6J	Probiotics		BDL + LGG (7) *vs.* BDL (7)	*In vitro* assay	/	[37]

a ↑, gut BSH activity was higher in the disease state or before intervention; ↓, gut BSH activity was lower in the disease state or before intervention. The numbers in the “Group” column indicate the total number of participants or animals per group. BSH: Bile salt hydrolase; KEGG: kyoto encyclopedia of genes and genomes; IsA: ilexsaponin A1; LGG: *Lactobacillus rhamnosus GG*; HC: health control; HFD: high-fat diet; CN: control; CAPE: caffeic acid phenethyl ester; PCPE: Penthorum chinense Pursh. extract; ALDH2-/--MCD & WT-MCD: ALDH2 knockout mice and wild-type littermate mice were fed a methionine-and choline-deficient diet; OCA: obeticholic acid; HFHC: high-fat and high-cholesterol diet; HF: high fat diet; TAA: thioacetamide; DDC: 3,5-diethoxycarbonyl-1,4-dihydrocollidine; KH: Kuhuang treatment; BDL: bile duct ligation; AAA-10: small-molecule inhibitor of gut bacterial BSHs.

### MASLD

Both clinical and animal studies consistently report a significant reduction in microbial BSH in MASLD. Two clinical studies from the USA and China showed that a significantly reduced abundance of BSH genes in the gut microbiome of MASLD patients^[9]^. Additionally, a metagenomic study in children with MASLD reported that the conversion of PBAs to SecBAs was inhibited, which was supported by a marked decrease in BSH-expressing genera (*Bacteroides* and *Eubacterium*)^[25]^. Numerous animal studies have further validated the reduction of BSH in MASLD mouse models. Modulating BA metabolism by targeting BSH-expressing bacteria has shown beneficial effects on cholesterol, triglyceride, and glucose levels. For instance, increasing the abundance of BSH-producing bacteria via herbal medicine elevated the levels of unconBAs in the serum and liver. This, in turn, upregulated the expression of Ehhadh and Hadha genes, promoting fatty acid degradation, lowering cholesterol and triglyceride levels, and improving glucose homeostasis^[26]^. He *et al.* demonstrated that a high-fat diet disrupted glucose homeostasis in mice and led to a reduction in hyocholic acid (HCA), which was associated with decreased abundance of *Lactobacillus* and *Bifidobacterium*, two key BSH-producing genera^[22]^. HCA is known to improve glucose homeostasis via distinct TGR5 and FXR signaling pathways^[27]^. In addition to HCA, other unconBAs such as ursodeoxycholic acid (UDCA) and LCA have also been found to offer metabolic benefits, including anti-inflammatory, antioxidant, and gut barrier-protective effects. Moreover, enhancing microbial BSH expression or activity can increase the ratio of unconBAs to conBAs. Because unconBAs are more hydrophobic, they are more readily excreted via feces. This increases hepatic BA synthesis, particularly via alternative pathways, thereby promoting cholesterol consumption and alleviating hepatic steatosis^[28,29]^.

However, not all findings are consistent. Some studies have reported increased microbial BSH expression in MASLD. In these cases, reducing BSH expression or suppressing BSH activity was found to increase the conBA/unconBA ratio, reduce cholesterol levels, and limit lipid accumulation. Elevated microbial BSH activity may raise concentrations of certain cytotoxic unconBAs, such as glycodeoxycholic acid, 7-ketodeoxycholic acid, and dehydrocholic acid, which can exacerbate liver damage^[11]^. Smirnova *et al.* reported significantly higher BSH gene expression in MASLD patients compared to healthy individuals, along with increased expression of dehydratase and HSDH^[11]^. These enzymes are essential for the production of DCA, which has been positively associated with MASLD severity. Conversely, reducing BSH expression may elevate the proportion of certain conBAs, such as tauro-β-muricholic acid (T-β-MCA), which inhibits intestinal FXR signaling, thereby enhancing the hepatic BA synthesis and reducing cholesterol levels^[17,30]^. Li *et al.* further observed that conBAs can form micelles with unconBAs, thereby sequestering unconBAs away from intestinal epithelial cells, reducing intestinal permeability damage, and mitigating hepatic inflammation^[31]^.

### MASH

MASH represents a more severe and progressive form of MASLD, characterized by liver inflammation and hepatocellular damage, and is closely associated with an increased risk of liver fibrosis. Although research on changes in BSH activity in MASH is limited, existing studies suggest that BSH expression is closely related to the metabolic functions of specific bacterial taxa. These bacteria utilize BSH to modulate the composition and concentration of the BA pool, particularly unconBAs. For example, reduced BSH expression in MASH mice has been associated with a decreased abundance of *Lactobacillus* in the gut microbiome. Supplementation with *Lactobacillus* has been shown to promote the formation of LCA, which can activate the FXR signaling pathway, thereby improving liver inflammation and fat accumulation^[32]^.

Conversely, other studies have reported a significant increase in BSH-expressing bacteria, such as *Bacteroides, Clostridium*, and *Lactobacillus*, in MASH mice fed a high-fat, high-cholesterol diet. This increase was positively correlated with elevated levels of unconBAs in the liver. Among these, excessive levels of DCA and chenodeoxycholic acid (CDCA) were found to significantly upregulate the expression of pro-inflammatory cytokines, including tumor necrosis factor-α (TNF-α) and interleukin-1β (IL-1β), exacerbating inflammation and hepatic steatosis^[33]^. Furthermore, the enrichment of *Bacteroides* has been linked to enhanced hydrolysis of TconBAs, resulting in the overproduction of CDCA in mice fed a Western diet. This, in turn, induces mitochondrial reactive oxygen species (ROS) accumulation and lipid peroxidation in the liver in a dose-dependent manner^[34]^.

### Liver fibrosis and cirrhosis

As the disease progresses, excessive extracellular matrix accumulation can drive the progression of MASH to advanced liver fibrosis and ultimately cirrhosis. Existing studies have consistently reported decreased microbial BSH gene expressions in liver fibrosis, leading to a significant accumulation of conBAs in the intestine. This accumulation may inhibit the FXR/FGF-15 signaling pathway, thereby aggravating liver damage^[19]^. Notably, the enrichment of BSH-expressing genera such as *Erysipelotrichaceae, Lactobacillus, Clostridium,* and *Bifidobacterium*^[35,36]^, as well as probiotic interventions with BSH-active strains^[18,37]^, has shown beneficial effects in reversing liver fibrosis. For example, one study reported that the fecal CDCA/taurochenodeoxycholic acid (TCDCA) ratio was significantly lower in patients with liver fibrosis compared to healthy individuals, suggesting reduced microbial BSH activity^[18]^. The study also observed a marked decrease in *Parabacteroides distasonis*, a bacterium known for high BSH expression. Supplementation with *P. distasonis* alleviated liver fibrosis by enhancing BSH activity and promoting TCDCA hydrolysis, thereby reducing hepatotoxicity. Similarly, probiotic treatment with *Lactobacillus rhamnosus GG* (LGG) demonstrated therapeutic effects in murine models of liver fibrosis^[37]^. LGG supplementation increased microbial BSH activity, facilitating BA deconjugation and promoting BA excretion via feces and urine, finally preventing BA-induced liver fibrosis by enhancing intestinal FXR-FGF-15 signaling. Additionally, LGG promoted the deconjugation of T-βMCA, mitigating its antagonistic effect on FXR and FGF-19 pathways. A large-scale cohort study further confirmed that BSH gene expression in the gut microbiota was significantly reduced in patients with cirrhosis^[38]^. These findings highlight the therapeutic potential of BSH-active probiotics in advanced liver diseases. Future studies should explore how BSH modulates the BA pool and related signaling pathways, which may lead to novel targeted strategies for early diagnosis and treatment of liver fibrosis.

### Causes of contradictory changes in BSH

The inconsistent findings regarding BSH activity in MASLD may be attributed to several factors. First, clinical and animal studies differ in sample sources, detection methods, and disease modeling approaches. Second, the heterogeneity of MASLD itself may lead to dynamic changes in BSH activity among different populations. Third, BSH-expressing bacteria may exhibit strain-specific functional differences; for example, *Lactobacillus* and *Bacteroides* strains may behave differently in the context of MASLD^[8]^. Song *et al.* reported that while atherosclerotic patients exhibited reduced total BSH abundance in the gut microbiota, the relative abundance of high-activity BSH subtypes was significantly increased, whereas low-activity subtypes were decreased^[16]^. Lastly, structural variations and substrate specificity among microbial BSH enzymes may produce divergent effects on BA metabolism (i.e., the relative composition and absolute concentration of both conBAs and unconBAs). For example, several studies have reported an increase in pro-inflammatory DCA and a decrease in hepatoprotective UDCA in MASLD. To summarize, these contradictory findings underscore the complexity of microbial BSH’s role in MASLD development and the need for further mechanistic research to clarify its function and therapeutic potential.

## BIOLOGICAL MECHANISMS UNDERLYING BACTERIAL BSH-DRIVEN THERAPEUTIC INTERVENTIONS

**Figure 1:**
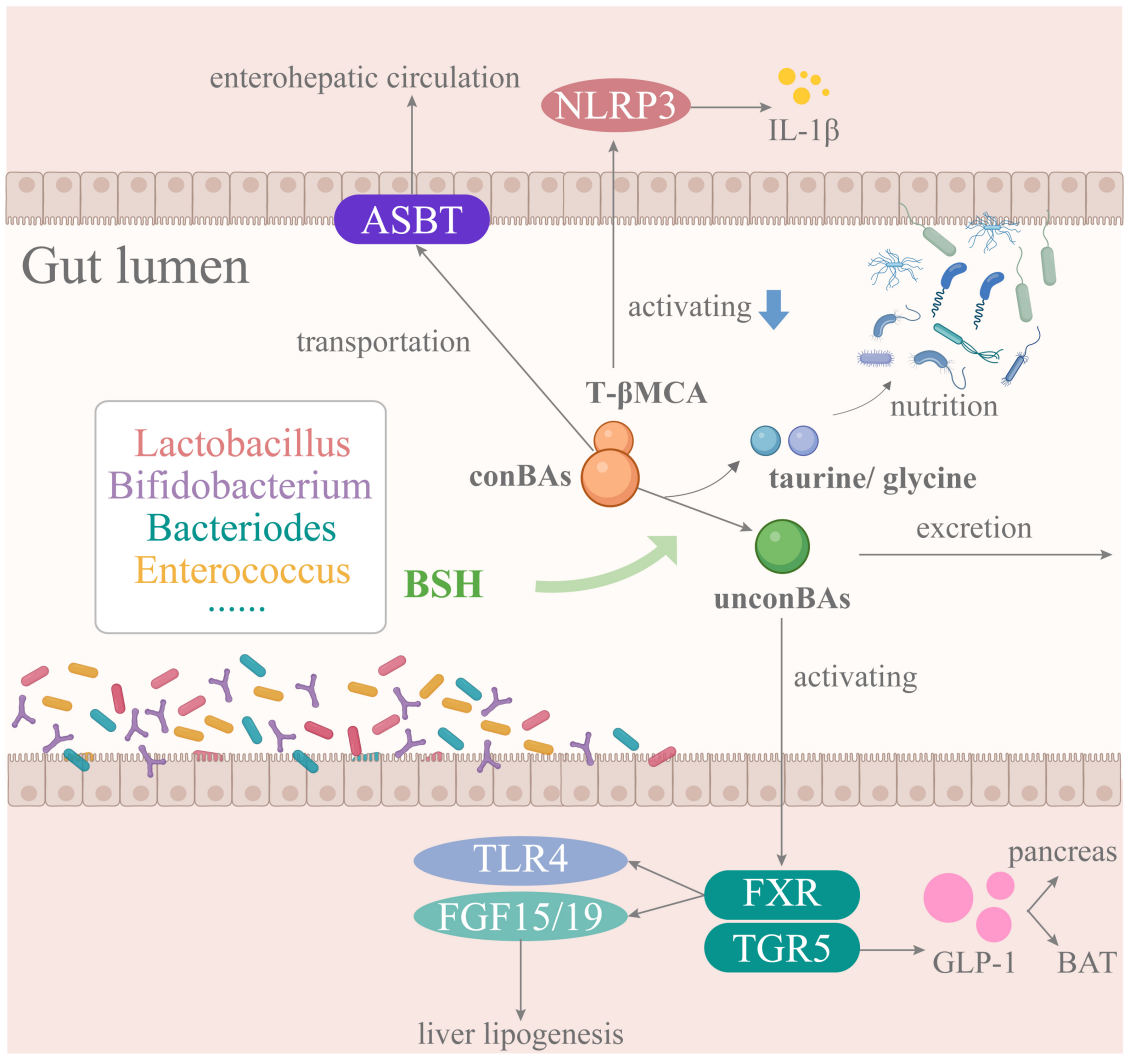
Potential therapeutic role of targeting microbial BSH expression in MASLD. MASLD: Metabolic dysfunction-associated steatotic liver disease; BSH: bile salt hydrolase; T-β-MCA: tauro-β-muricholic acid; FXR: farnesol X receptor; conBAs: conjugated bile acids; unconBAs: unconjugated bile acids; BAT: brown adipose tissue.

This section summarizes three main biological mechanisms through which targeting microbial BSH may contribute to the prevention and treatment of MASLD [Figure 1].

### Microbial BSH-mediated modulation of BA metabolism

Regulating BA deconjugation through BSH represents a promising therapeutic approach for managing MASLD. Probiotics, prebiotics, and BA sequestrants have shown potential in modulating BA metabolism and thereby improving liver health in MASLD patients^[39]^. Compared to conBAs, unconBAs are more hydrophobic and less soluble, facilitating their excretion or utilization by gut bacteria^[40]^. This process triggers a feedback mechanism that stimulates the synthesis of primary BAs, thereby enhancing cholesterol utilization^[41]^. Probiotics offer unique advantages in MASLD management, with BSH activity playing a crucial role in their colonization and growth in the gastrointestinal tract^[42]^. Several well-characterized BSH-active probiotic strains, primarily *lactobacilli* derived from both human and non-human origins, exhibit cholesterol-lowering effects and show promise for clinical application^[43]^. Notably, *Lactobacillus plantarum* strains Lp91 and Lp21 demonstrate strong BSH activity, with a substrate preference for glycocholate over other amino acid conjugates^[44]^. Moreover, colonization by BSH-positive bacteria is essential for BA hydrolysis and subsequent re-amidation^[15]^. Genetic manipulation of these strains - either via BSH gene deletion or overexpression - can influence BA synthesis and conversion, offering therapeutic potential for modulating inflammation and liver diseases^[14]^. BSH hydrolyzes conjugated BAs, thereby reducing their toxicity to bacterial cell membranes, while the released taurine or glycine can be utilized as an energy source, conferring a nutritional advantage to the bacteria^[45]^. Finally, diet interventions or bioactive compounds may benefit MASLD through their influence on microbiota-BA interactions. For instance, Zhao *et al.* reported that administration of ilexsaponin A1 (IsA) significantly improved insulin resistance and hepatic steatosis in MASLD mice^[28]^. This improvement was linked to increased BSH activity in the gut microbiome, which promoted conjugated BA hydrolysis and reduced hepatic BA accumulation.

### Modulation of FXR and TGR5 signaling pathways to improve glucose and lipid metabolism

FXR and TGR5 are two key BA receptors involved in regulating energy expenditure, reducing inflammation, and maintaining gut barrier integrity, making them attractive therapeutic targets for MASLD^[7]^. Microbial BSH influences the composition and concentration of secBAs, which can in turn modulate FXR and TGR5 signaling pathways.

Importantly, FXR exhibits tissue-specific effects - acting differently in the liver and intestine. Intestinal BSH inhibition can lead to the accumulation of endogenous FXR antagonists, thereby helping to restore metabolic balance in the gut. Several studies have reported that BSH inhibitors^[17]^, antioxidants^[46]^, and natural compounds^[47]^ can reduce microbial BSH expression and/or activity in mouse models, resulting in increased levels of T-β-MCA, an endogenous FXR antagonist. This suppression of FXR signaling in ileal epithelial cells reduces ceramide synthesis and promotes GLP-1 secretion, ultimately improving metabolic outcomes. Conversely, supplementation with BSH-active *Lactobacillus plantarum* increases CDCA levels, which suppress hepatic lipogenesis and insulin resistance^[48]^ via FXR signaling. Activation of hepatic FXR inhibits triglyceride production by downregulating the SREBP-1c lipogenesis pathway^[49]^. Additionally, FXR agonists such as obeticholic acid have been shown to promote brown fat differentiation and energy metabolism^[50]^.

TGR5 also plays an important role in glucose and lipid metabolism and in mitigating inflammation in MASLD. Bariatric surgery, known for its metabolic benefits, increases levels of intestinal and circulating taurine-conjugated BAs, which in turn activate FXR and TGR5 signaling, thereby stimulating adaptive thermogenesis^[51]^. Salidroside has been found to alleviate lipid accumulation and inflammatory injury in MASH mice by increasing the abundance of BSH-expressing bacteria and decreasing conjugated BA levels, thereby enhancing downstream FXR and TGR5 activation^[52]^. Interestingly, oral administration of live *Parabacteroides distasonis* has been shown to alleviate inflammatory arthritis. This effect was attributed to its BSH-derived metabolites, 3-oxoLCA and isoLCA, which act as TGR5 agonists and promote M2 macrophage polarization^[53]^.

### Inhibiting NLRP3 to alleviate inflammation

The NLR family pyrin domain containing 3 (NLRP3) inflammasome is an intracellular sensor implicated in the pathogenesis of various metabolic diseases, including MASLD. BAs can dose-dependently activate the NLRP3 inflammasome, leading to IL-1β secretion and liver fibrosis^[54]^. This activation occurs through mechanisms such as potassium efflux and ROS generation. In mouse models, reduced BSH activity lowers levels of secondary BAs like nor-deoxycholic acid (NorDCA), which in turn suppresses NLRP3 activation and mitigates liver inflammation^[55]^. Sun *et al.* found that *Bacteroides dorei BDX-01* enhanced BSH protein expression in the intestines of mice, increasing the β-MCA to T-β-MCA ratio and decreasing NLRP3 and IL-1β expression in the colon^[56]^. These changes significantly alleviated intestinal inflammation. Notably, T-β-MCA antagonized the inhibitory effect of BDX-01 on NLRP3 inflammasome activity *in vitro*. Thus, modulating BSH activity alters the composition of secondary BAs, which can impact NLRP3 inflammasome activation and inflammation. Among these, T-β-MCA appears to be a particularly noteworthy target molecule.

## CHALLENGES AND FUTURE DIRECTIONS

As the “gatekeeper” responsible for initiating secondary modifications of BAs in the gut, BSH plays a pivotal role in linking the gut microbiome with BA metabolism. Although no consensus has been reached regarding the precise pattern of BSH changes in individuals with MASLD, it is evident that such changes are closely related to disease progression. Most studies have demonstrated that intestinal BSH expression or activity tends to decline in more advanced stages of MASLD, such as the onset of hepatitis or fibrosis. These changes primarily affect the ratio of conjugated to unconjugated BAs, thereby disrupting the dynamic balance of the BA metabolic network. This, in turn, influences enterohepatic circulation and various metabolic signaling pathways.

### Challenges in evaluating BSH activity in MASLD

Several factors contribute to the current uncertainties regarding the role of BSH in MASLD progression. First, the degree of disease severity varies among individuals, leading to differences in gut microbiome composition and BA profiles. Clinical studies have shown that MASLD patients with steatohepatitis exhibit significantly elevated serum levels of taurocholic acid (TCA), glycocholic acid (GCA), and taurolithocholic acid (TLCA) compared to those with simple steatosis^[57]^. The worsening of hepatic steatosis, inflammation, and ballooning degeneration is closely linked to the increase in these conBAs. Second, because BAs serve diverse biological functions, microbial BSH directly influences the levels of both beneficial and harmful secondary BAs. Consequently, studies focusing on specific BAs may yield varying conclusions regarding the relationship between BSH activity and disease progression. Third, the BA pool in mice contains a higher proportion of primary BAs and a broader range of secondary BAs than in humans^[5]^, leading to potential discrepancies in BSH-related findings across species. Lastly, different methodologies have been employed to measure BSH expression and activity, with varying levels of sensitivity and accuracy, making direct comparison across studies challenging.

### Therapeutic implications and potential interventions targeting BSH

Studying the role of microbial BSH in host metabolism presents methodological challenges due to the variability in gut microbiota composition and the complexity of BA metabolism. Longitudinal studies are therefore necessary to understand the long-term effects of microbial BSH on MASLD progression and to identify reliable biomarkers for disease monitoring. In addition, personalized approaches that account for individual differences in gut microbiota and metabolic responses will be critical for the development of effective BSH-targeted therapies.

Recent advances in microbiome research, metagenomics, and metabolomics are opening new avenues for understanding the role of BSH in MASLD and for developing targeted interventions. BSH not only supports bacterial colonization and survival in the gastrointestinal tract but also significantly affects host glucose and lipid metabolism and the regulation of inflammation. Its regulatory effects are mediated through BAs, especially via alterations in the composition and spatial distribution of secondary BAs, which interact with specific receptors and the NLRP3 inflammasome. These interactions indirectly influence the synthesis and secretion of key metabolic regulators such as cholesterol, ceramides, and GLP-1, contributing to the restoration of metabolic balance. This highlights the potential of BSH as a therapeutic target for MASLD. Consequently, the use of probiotics or natural small-molecule compounds to modulate microbial BSH gene expression or enzyme activity has become a focal point of research aimed at improving MASLD-related metabolic disorders^[28]^.

Although extensive studies have explored the structure, function, and substrate specificity of various BSH enzymes, much remains to be discovered about gut bacteria and their functions^[58]^. With ongoing advancements in sequencing technologies, additional BSH variants are expected to be identified. Understanding the enzymatic properties of these variants will be crucial for developing personalized probiotics and more effective diagnostic and therapeutic tools.

## CONCLUSION

Gut microbial BSH activity tends to decline during the development and progression of MASLD due to factors such as changes in gut microbiota composition, host metabolic status, inflammation, and dietary influences. BSH plays a critical role in maintaining BA metabolic balance and significantly impacts the progression and management of MASLD. A deeper understanding of the intricate relationship between gut microbiota, BSH, and MASLD will provide valuable insights for the development of targeted therapeutic strategies. Further research is needed to clarify the mechanisms by which BSH changes influence liver health and to devise effective interventions for MASLD. Targeting BSH represents a promising approach for modulating BA metabolism and improving metabolic health, highlighting the crucial role of the gut microbiota in disease management.

## DECLARATIONS

### Copyright

© The Author(s) 2025.
